# Efficacy and safety of ultrasound-guided peripheral nerve blocks in management of chronic resistant migraine

**DOI:** 10.1186/s10194-025-02013-3

**Published:** 2025-04-16

**Authors:** Nourhan Abdelmohsen Taha, Mai Fathy, Ahmed Elsadek, Tamer Hussein Emara, Sherien Mohamed Farag, Ramez Reda Moustafa, Mohamad Osama Abdulghani

**Affiliations:** Department of Neurology, Faculty of Medicine, Ain Shams University, 38 Abbasia, PO 11591, Cairo, Egypt

**Keywords:** Chronic migraine, Resistant migraine, Occipital nerve, Nerve block, Sphenopalatine ganglion, Autonomic manifestations, Egypt

## Abstract

**Background:**

Migraine is a common primary headache disorder with different treatment modalities emerging as ultrasound guided peripheral nerve blocks. We compared the efficacy and safety of ultrasound guided bilateral sphenopalatine ganglion (SPG) block versus bilateral greater occipital nerve (GON) block, in chronic resistant migraine patients and controls.

**Methods:**

This study was an interventional randomized controlled trial, including 53 patients, 22 in sphenopalatine ganglion arm, 21 greater occipital nerve arm and 10 in sham group. All patients were assessed initially by headache diary (for 3 months), HIT-6 and MIDAS scales. The patients (blindly allocated) underwent nerve block ultrasound guided, then followed up after one month by headache diary and HIT- 6 scale and three months by MIDAS. Results were analysed on SPSS, using mixed AVOVA and Tukey’s Post-Hoc analysis, Fisher’s exact and paired t-test.

**Results:**

The two groups were matched as regards the gender, age, type of migraine, frequency and years lived with headache. The study revealed that GON and SPG block, were equally effective (*p* < 0.05) as regards reducing the headache diary parameters, as well as the total pain index and the functional impact on HIT-6 and MIDAS scale. SPG block was more effective in patients with autonomic manifestations and temporal location of pain.

**Conclusion:**

Ultrasound guided SPG is as effective as GON as a treatment modality for chronic resistant migraine and may be more useful in the presence of autonomic manifestations and temporal location of pain.

## Background

Peripheral nerve blocks have evolved over the past decades as an effective line of treatment for various types of resistant and refractory headaches and is increasingly being used among headache specialists. They are considered a safe and effective alternative to conventional medical treatment in patients with significant co-morbidities, or when use of medical treatment isn’t safe or limited by pregnancy or lactation and in those intolerant to the side effects [1].

Studies on nerve block in migraine often involve chronic migraine (CM) (> 15 headache days/month, 8 of which are migraine, for 3 months), and its more challenging subtype, chronic resistant migraine, defined according to the European headache federation as resistance or intolerance to three lines of effective migraine treatment [2].

The most studied nerve block for chronic migraine (CM) is that of the greater occipital nerve (GON) [3]. The GON originates from the dorsal rami of the 1st three cervical roots, relaying at the spinal nucleus of trigeminal forming cervical-trigeminal complex. Recently, a meta-analysis showed that GON intervention could significantly reduce pain intensity and analgesic medication consumption in migraine [4] and another study showed significant reduction in calcitonin gene related peptide (CGRP) levels among migraine patients compared to placebo [5]. Ultrasound guided GON block at the level of C2 in comparison to distal GON block shows a higher response rate in terms of the number of severe attacks and analgesic consumption [6].

Another commonly studied nerve block is that of the sphenopalatine ganglion (SPG), as the SPG is a major relay station in the trigemino-vascular network that mediates the autonomic activity associated with migraine along with vasodilatation of the meningeal blood vessels [7]. SPG block was shown to be effective for the chronic resistant migraine, yet most studies assessed its efficacy through trans-nasal approach or supra-zygomatic injection in acute management [8, 9].

Percutaneous infra-zygomatic approach using fluoroscopy-guided needle placement has been used for direct administration of the drugs to the SPG rather than diffusion across mucous membranes as in the trans-nasal approach [10]. The most common indication for this procedure has been cluster headache [11]. The use of ultrasound-guided techniques instead of fluoroscopy-based imaging, is considered a safer alternative and with no need for contrast injection [12].

In this study we compared the efficacy and safety of ultrasound guided infra-zygomatic SPG block to that of ultrasound-guided proximal GON block in chronic resistant migraine patients and controls.

## Methods

This study was a randomized controlled trial, recruiting patients attending the headache clinic at Ain Shams University hospitals (A tertiary hospital serving Eastern Greater Cairo) from March 2022 till March 2024.

The patients recruited were > 18 years of age with a diagnosis of chronic resistant migraine (suffering > 15 headache days/month, 8 of which are migraine, for 3 months, being resistant or intolerant to three lines of effective migraine treatment, according to EHF consensus criteria [2]). We excluded patients who underwent previous nerve blocks or had bleeding tendency or infection at site of injection and patients with any CNS disorders causing brain lesions, or presence of other types of headaches, or history of significant head trauma.

### Study procedures

All eligible patients were interviewed with a semi-structured questionnaire for the age, gender, and years lived with headache. Patients underwent full neurological examination as well as fundus assessment to rule out idiopathic intracranial hypertension being a cause for resistant migraine.

Patients were initially assessed by headache diary (over past 3 months) as regards the frequency, character and duration of the headaches, and the pattern of usage of analgesics, the number of tablets used/day and number of days/month and the severity of pain by NRS-11 [13],. Total Pain Burden (TPB) score was calculated as the product of the frequency, duration, and intensity of the attacks [14]. The impact of headache on functionality and quality of life was assessed by HIT-6 [15] and MIDAS Arabic version [16, 17] scales. The presence or absence of cranial autonomic symptoms was recorded. Up to 70% of chronic migraine patients have autonomic manifestations similar to those of autonomic cephalalgias such as lacrimation, eye congestion, ptosis, facial sweating, nasal congestion and rhinorrhea [18].

Patients underwent SPG block (group 1), GON block (group 2) or sham SPG injection (group 3) only once during the study. The procedures were performed by well-trained neurologists.

**Fig. 1:**
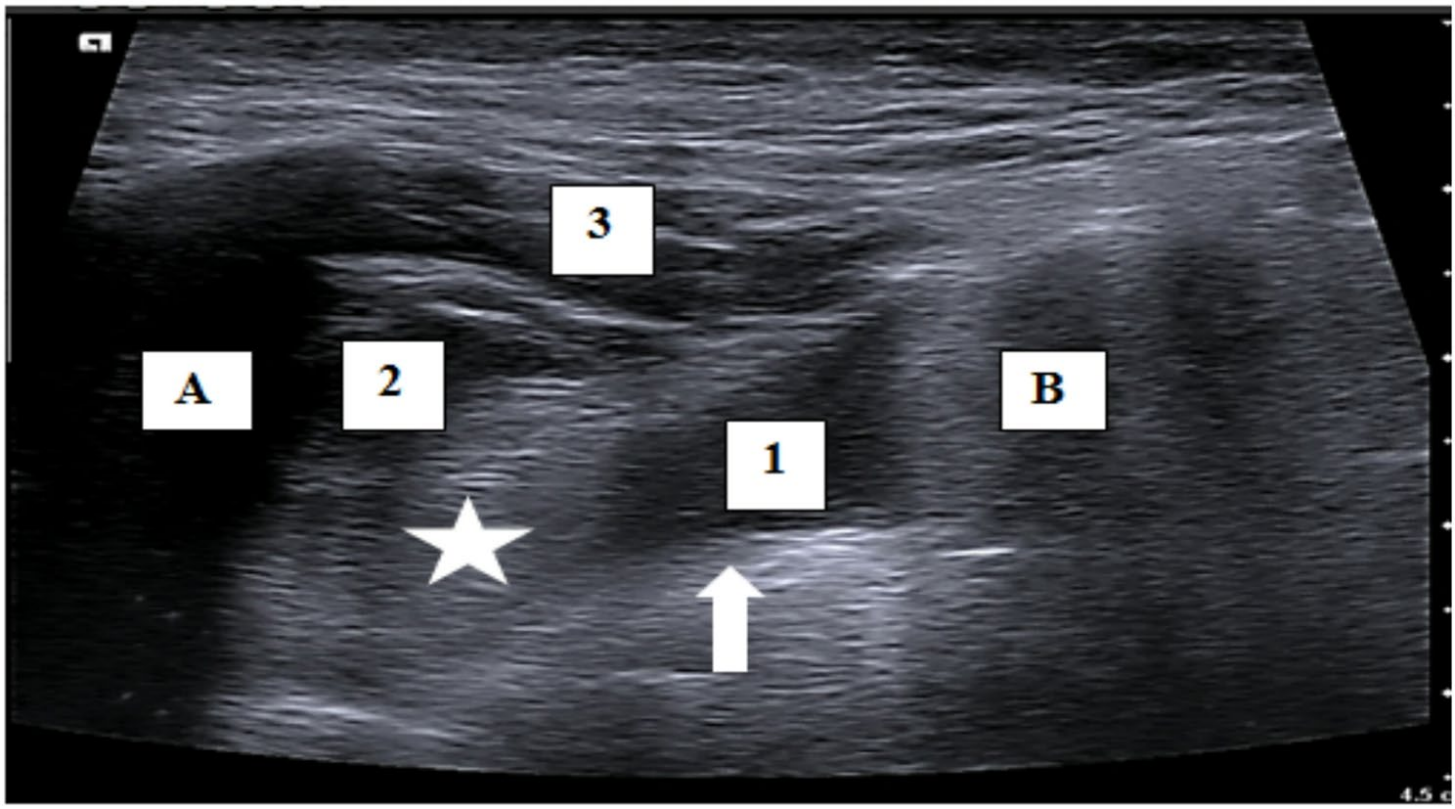
Ultrasound Image of the Sphenopalatine fossa anatomy. Anatomy of the sphenopalatine fossa as seen by ultrasound transverse plane infra-zygomatic approach. (live image). (A): The Coronoid process, (B): The Condylar process, (1): lateral pterygoid muscle, (2): temporalis muscle, (3) masseter muscle, (arrow): the lateral pterygoid plate, (star): is the target point (SPG)

Ultrasound-guided SPG block was done via the lateral infra-zygomatic approach with a linear 4-12 MHz probe (Esaote My Lab Five, Italy) adjusted at lowest frequency, to visualize the configuration of the sphenopalatine fossa. The lateral pterygoid plate (a hyperechoic transverse line) form the floor, the mandibular processes anteriorly and posteriorly (hypoechoic vertical shadows) and through the mandibular notch the ultrasound waves penetrate to visualize the lateral pterygoid muscle (a triangular hypoechoic shadow overlying the pterygoid plate), the deep head of masseter (lying superior and anterior to pterygoid muscle) with the sphenopalatine ganglion located at the anterior apex of the lateral pterygoid muscle (Fig. 1). The patient was placed in supine position, the mandibular notch was identified by asking the patient to open his mouth while palpating the area just anterior and inferior to the acoustic auditory meatus. Once identified, the probe was placed transversely over the notch to visualize the sphenopalatine fossa, and the patient was asked to hold his mouth open. The vessels were visualized by colour mode to avoid any vascular injury and using a 22-gauge spinal needle, the sphenopalatine ganglion was approached by out-of-plane technique. 1 ml of lidocaine was injected subcutaneously at the injection site using U-100 31G needle as a local anaesthetic. Patients were injected ***on each side*** by either long-acting steroids triamcinolone or betamethasone, along with 1 ml of lidocaine 2%, with total amount of injected fluids being 4 ml on each side.

We added corticosteroids to the local anaesthetic based on the hypothesis that it contributes a potential anti-inflammatory effect related to the release of inflammatory cytokines following trigeminal activation. Meta-analyses have reported that while some studies found no significant benefit, others demonstrated an additional clinical advantage when steroids were combined with local anaesthetics [4, 19].

At our centre, no serious adverse events related to either particulate and non-particulate steroid use have been reported in similar procedures. Additionally, we took several precautions to minimize the risk of vascular injury, including the use of colour Doppler imaging during the injection to identify and avoid nearby vessels, as well as performing negative aspiration prior to injection to reduce the risk of intravascular administration. Moreover, previous studies have reported the use of triamcinolone in similar settings without significant adverse events, which further supported our decision to include it in our protocol [20].

**Fig. 2:**
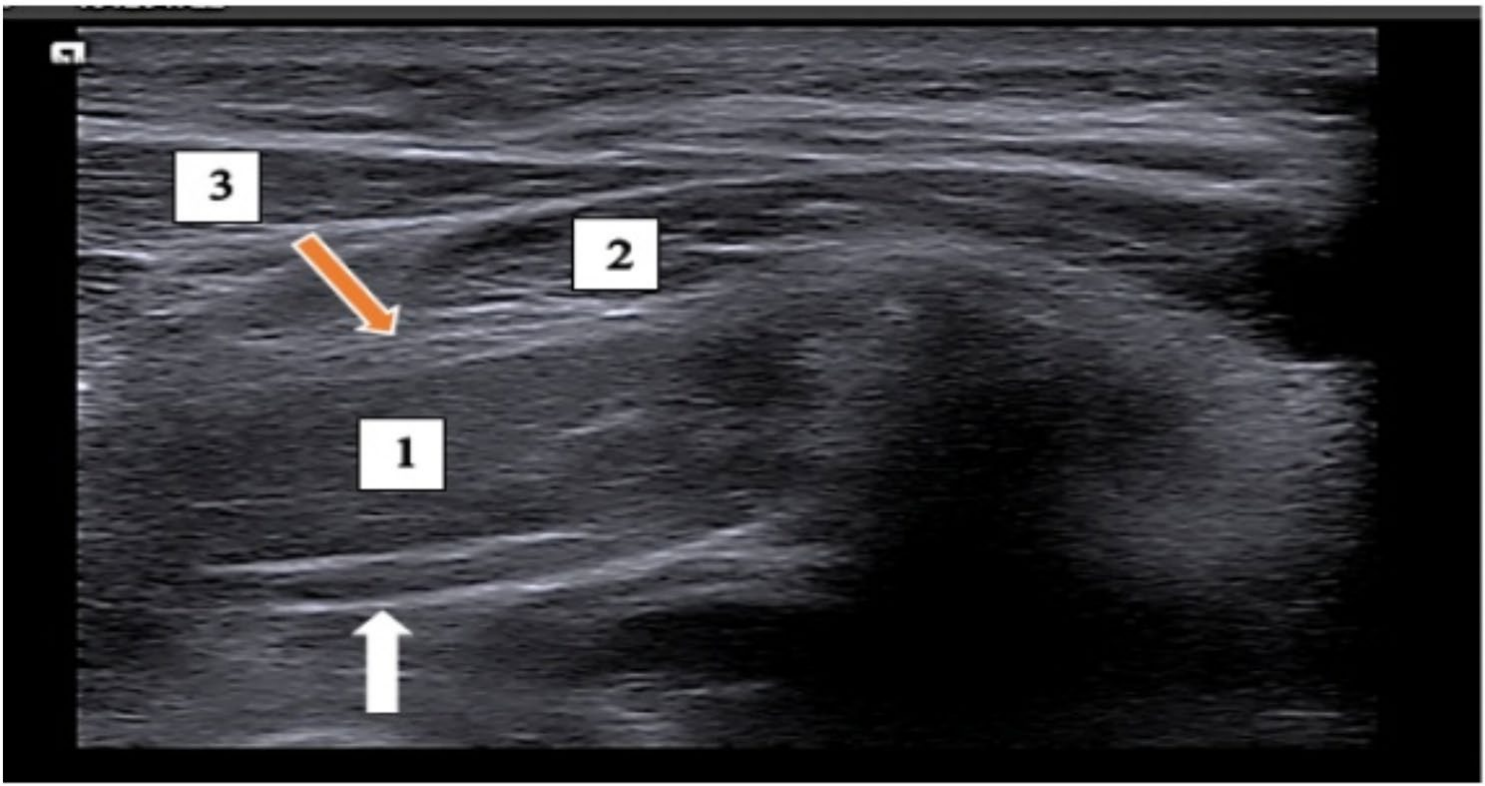
Ultrasound image of greater occipital nerve anatomy. Anatomy of the greater occipital nerve as illustrated by ultrasound transverse plane at level of C2 (live image). 1: Obliqus capitis muscle, 2: semispinalis capitis muscle, 3: splenius capitis muscle, white arrow: the lamina of C2, orange arrow: the fascia containing the GON and artery (target point)

Ultrasound-guided GON block was performed using the same probe and ultrasonography device, where the probe was placed at the superior nuchal line, at level of C2, visualizing a vertical hypoechoic shadow of C2 transverse process and from deep to superficial the following layers were identified; C2 lamina (hyperechoic horizontal line), Obliqus capitis and semispinalis capitis muscles, with splenius capitis and trapezius being most superficial. The fascial thickness between the obliqus capitis inferiorly and the semispinalis capitis superiorly contained the greater occipital artery that was visualized as a pulsatile structure by B-mode and confirmed using colour doppler mode with the nerve located medial to the artery. Using a 3 ml 24-gauge needle, the GON was approached by in-plane technique from lateral to medial (Fig. 2) and patient was injected on each side using the same long-acting steroids and lidocaine mix.

For the sham group, patients were injected by normal saline using the same technique for SPG block.

The patients were reassessed after 1 month by the headache diary, NRS-11 and HIT- 6 scale and after 3 months by MIDAS, with maintaining the preventive medications fixed, either the type or dose, before and after the intervention.

The response of the patients in reduction of number of headache days per month was classified as good (> 50%), poor (< 50%) and no response.

### Statistical methods

The sample size was calculated using PASS 11.0 program (www.ncss.com/software/pass/) to be ≥ 50 patients in 3 groups; Group 1 (≥ 20 patients) and Group II (≥ 20 patients) and Sham group (≥ 10 patients) achieve 81% power with a significance level (alpha_ of 0.05000 using a two sided two-sample t-test). Simple Randomization method was used using the Research Randomizer software (Scott Plous, Wesleyan University, USA).

Statistical package for Social Science (SPSS 27, IBM, USA) was used for all analyses. Comparisons were done using mixed-model **ANOVA** test to assess the statistical significance of the difference between the three study groups. **Tukey’s Post Hoc Test** was used for comparisons of all possible pairs of group means. **Fisher’s exact test** was used to examine the relationship between two qualitative variables when the expected count was less than 5 in more than 20% of cells, and **Paired t-test** was used to assess the statistical significance of the difference between two means measured twice for the same study group. **Level of significance was set as ***p* > 0.05: Non-significant and *p < 0.05: Significant.*

## Results

Out of 250 patients screened, 80 cases were eligible, and 27 cases were excluded either due to refusal to participate in the study (*N* = 20) or due to narrow mandibular notch (*N* = 3), poor sonographic view (*N* = 3) and TMJ disorders (*N* = 1). The remaining 53 patients were randomly assigned to the 3 groups (22, 21 and 10 respectively).

As regards the descriptive characteristics of the study population there was no difference between groups as regards age, gender, diagnosis, and years lived with headache among the three groups (Table 1). There was also no difference between groups initially as regards the number of headache days per month, intensity on NRS scale and duration in hours of headache attacks, the medication overuse (number of days/month with analgesic consumption) or the functional impact by HIT-6 (Table 2).

**Table 1:** Descriptive analysis of the sociodemographic data and the clinical features of the recruited patients among each group

		Group			*P*
		Sham group (*N* = 10)	SPG group (*N* = 22)	GON group (*N* = 21)	
		Mean ± SD *N* (%)	Mean ± SD *N* (%)	Mean ± SD *N* (%)	
Age		35.2 ± 8.8	37.32 ± 10.74	30.62 ± 11.28	0.124
Years of headache		9.1 ± 4.86	6.25 ± 5.07	9.57 ± 10.24	0.326
Gender	Male	1 (10%)	3 (13.64%)	1 (4.76%)	0.831
	Female	9 (90%)	19 (86.36%)	20 (95.24%)	
Diagnosis	Chronic migraine without aura	9 (90%)	19 (86.36%)	17 (80.95%)	0.89
	Chronic migraine with aura	1 (10%)	3 (13.64%)	4 (19.05%)	
Site	Temporal		11 (50%)	11 (52.4%)	0.374
	Occipital		4 (18.2%)	7 (33.3%)	
	Temporal and occipital		7 (31.8%)	3 (14.3%)	
Side	Unilateral		2 (9.1%)	2 (9.5%)	1.00
	Bilateral		20 (90.9%)	19 (90.5%)	
Autonomic Manifestation	None		14 (63.6%)	19 (90.5%)	0.069
	Yes		8 (36.4%)	2 (9.5%)	

SPG: Sphenopalatine Ganglion, GON: Greater Occipital Nerve, SD: Standard Deviation

**Fig. 3:**
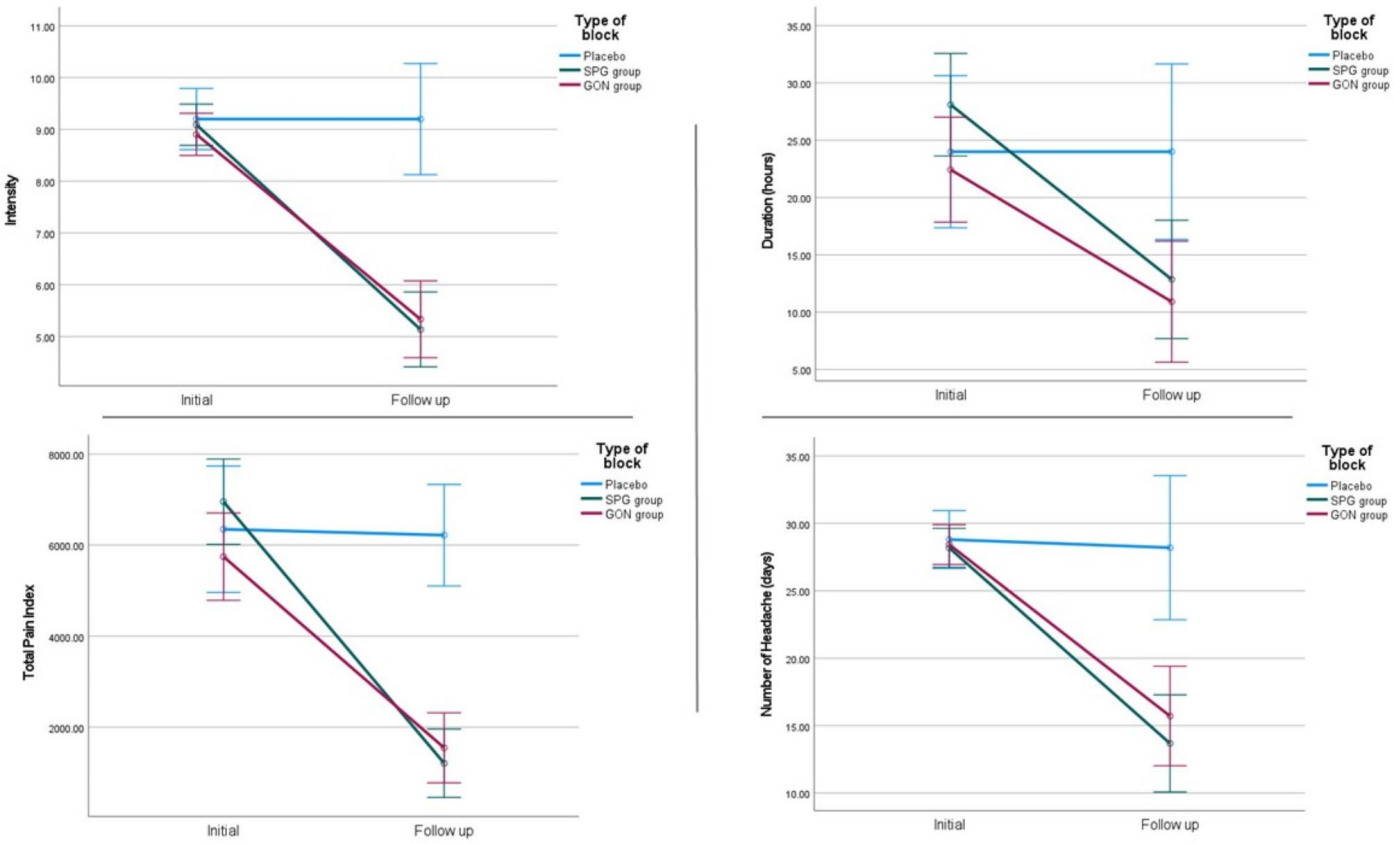
The change in headache diary parameters after nerve block in each study group. Reduction of headache frequency, intensity, duration, and total pain index in the active groups compared to placebo group (blue line), with non-significant difference between SPG (green line) and GON (red line) group

At 1 month, there was a statistically significant improvement in all the parameters of the headache diary in SPG and GON groups compared to sham group. Post-hoc analysis showed that there was, however, no difference between either type of block (Table 2) (Fig. 3).

**Table 2:** Comparative analysis of the different headache diary parameters and functional impact scores

		Group			One Way ANOVA	
		Sham group (*N* = 10)	SPG group (*N* = 22)	GON group (*N* = 21)		
		Mean ± SD	Mean ± SD	Mean ± SD	value	*p*-value
Number of Headache days	Initial	28.8 ± 1.07	28.18 ± 0.72	28.43 ± 0.74	0.117	0.890
	Follow up	28.2 ± 2.66	13.68 ± 1.79	15.71 ± 1.84	10.787	< 0.001
Pairwise comparison	p-value	0.82	< 0.001	< 0.001		
Duration in hours	Initial	24 ± 3.3	28.09 ± 2.23	22.43 ± 2.28	1.643	0.204
	Follow up	24 ± 3.81	12.86 ± 2.57	10.92 ± 2.63	4.224	0.020
Pairwise comparison	p-value	1.00	< 0.001	< 0.001		
Intensity	Initial	9.2 ± 0.29	9.09 ± 0.2	8.9 ± 0.2	0.403	0.670
	Follow up	9.2 ± 0.53	5.14 ± 0.36	5.33 ± 0.37	22.422	< 0.001
Pairwise comparison	p-value	1.00	< 0.001	< 0.001		
Total Pain Index	Initial	6348 ± 692.07	6954.55 ± 466.59	5746.29 ± 477.57	1.638	0.205
	Follow up	6218.4 ± 555.18	1207.68 ± 374.3	1547.1 ± 383.11	31.094	< 0.001
Pairwise comparison	p-value	0.859	< 0.001	< 0.001		
No. of Analgesic tablets	Initial	3.2 ± 0.62	4 ± 0.41	3.24 ± 0.42	1.019	0.368
	Follow up	3.2 ± 0.28	1.55 ± 0.19	1.19 ± 0.19	18.582	< 0.001
Pairwise comparison	p-value	1.00	< 0.001	< 0.001		
No. of days/month	Initial	28.8 ± 2	28.86 ± 1.35	25.95 ± 1.38	1.322	0.276
	Follow up	28.2 ± 2.74	9.23 ± 1.85	9.38 ± 1.89	19.264	< 0.001
Pairwise comparison	p-value	0.834	< 0.001	< 0.001		
MIDAS score in days	Initial	111.3 ± 17.19	108.27 ± 11.59	76.43 ± 11.87	2.316	0.109
	Follow up	111.3 ± 14.63	38.09 ± 9.86	30.14 ± 10.09	11.424	< 0.001
Pairwise comparison	p-value	1.00	< 0.001	< 0.001		
HIT-6 score	Initial	69.8 ± 1.34	72.91 ± 0.9	71.95 ± 0.92	1.862	0.166
	Follow up	69.8 ± 3.3	54.95 ± 2.23	54.48 ± 2.28	8.480	0.001
Pairwise comparison	p-value	1.00	< 0.001	< 0.001		

*One Way ANOVA test of significance (f) – Post-hoc analysis was significant between (Sham Vs. SPG and GON groups); ** paired t-test

SPG: Sphenopalatine Ganglion, GON: Greater Occipital Nerve, SD: Standard Deviation

**Fig. 4:**
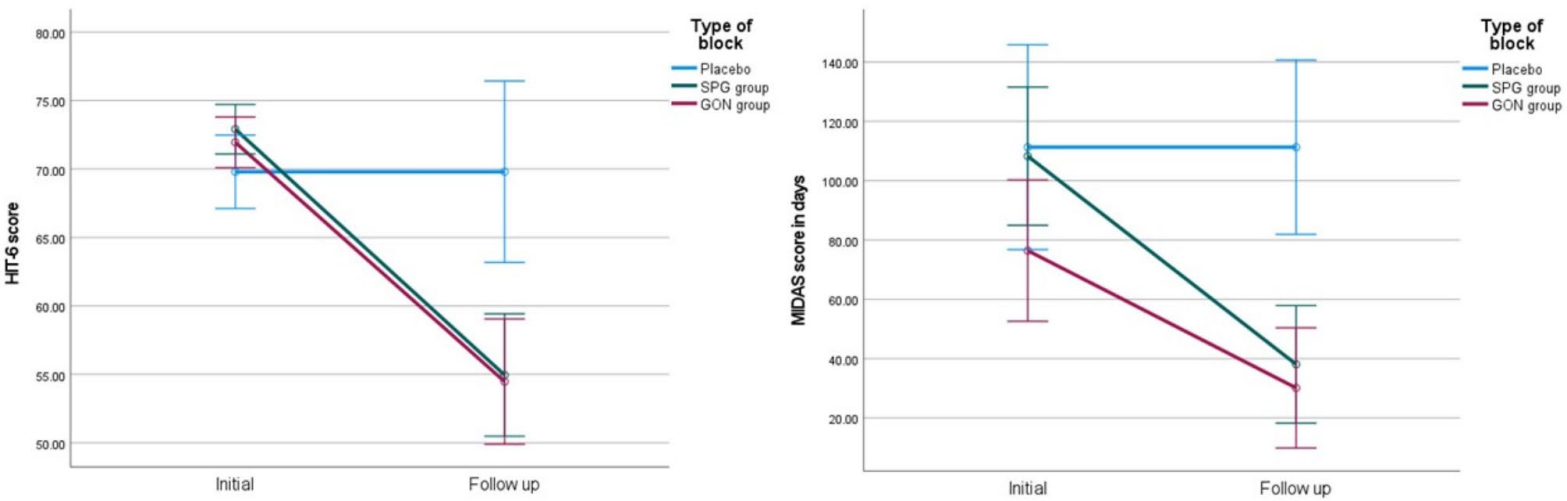
The change in functional impairment scales score after nerve block in each study group. Reduction in the scores of HIT-6 and MIDAS scales in the active groups compared to placebo group (blue line), with no significant difference between SPG (green line) and GON (red line) group

As regards the functional impact of migraine in the SPG and GON block groups compared to sham group, there was significant reduction in HIT-6 total score after 1 month (*p* < 0.001), yet post-hoc analysis showed absence of difference between either of the active groups (Table 2) (Fig. 4).

At 3 months, the MIDAS scale scores showed significant reduction in SPG group and GON group compared to sham group (*p* < 0.001), with non-significant difference between the active groups (Table 2) (Fig. 4).

**Fig. 5:**
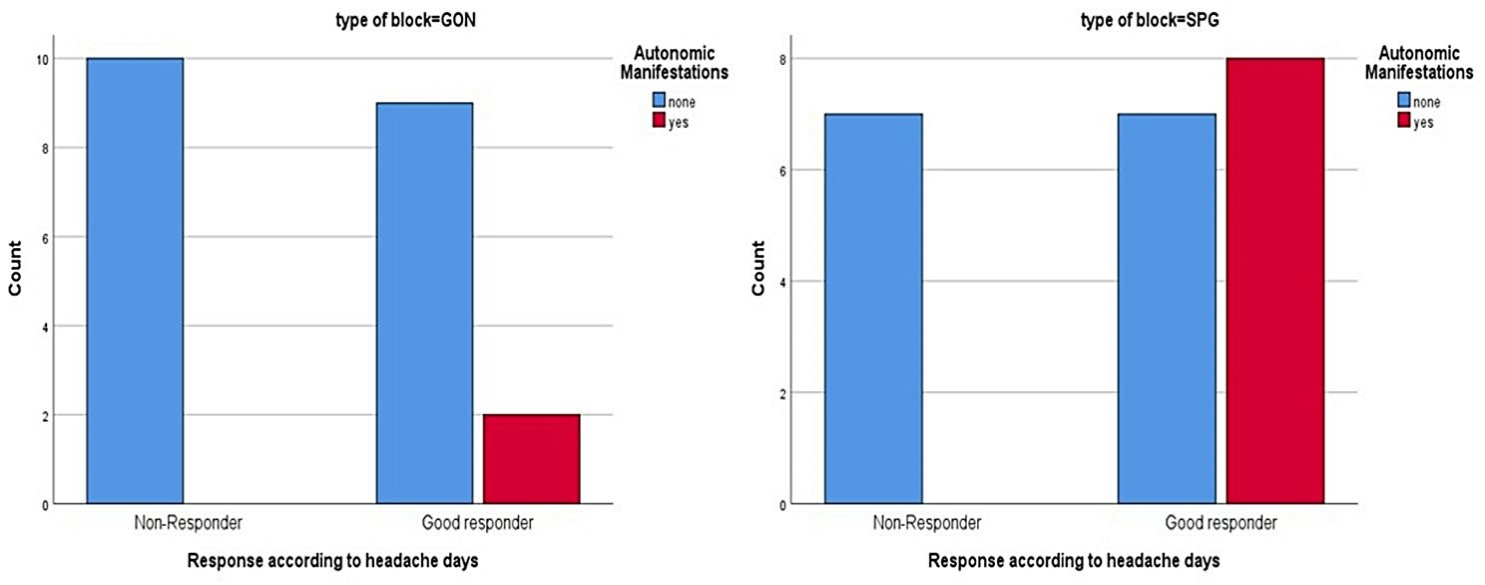
Responders rate among the two active groups in relation to autonomic manifestations. Difference in response according to headache days in the SPG group (right image) and GON group (left image) in relation to presence of autonomic manifestations

The presence of autonomic symptoms (lacrimation, nasal congestion, ear fullness and vertigo) favoured a better outcome in the SPG group, showing a statistically significant improvement compared to greater occipital block (Table 3) (Fig. 5).

**Table 3:** Comparison of the response in relation to autonomic manifestations among the two groups

Type of block	Response		Autonomic Manifestations		Fisher’s exact test
			None	Yes	P-value
GON	Response according to headache days	Non-Responder	10 (52.6%)	0 (0%)	0.262
		Good responder	9 (47.4%)	2 (100%)	
	Total		19 (100%)	2 (100%)	
SPG	Response according to headache days	Non-Responder	7 (50%)	0	0.020*
		Good responder	7 (50%)	8 (100%)	
	Total		14 (100%)	8 (100%)	

SPG: Sphenopalatine ganglion, GON: Greater Occipital nerve

**Fig. 6:**
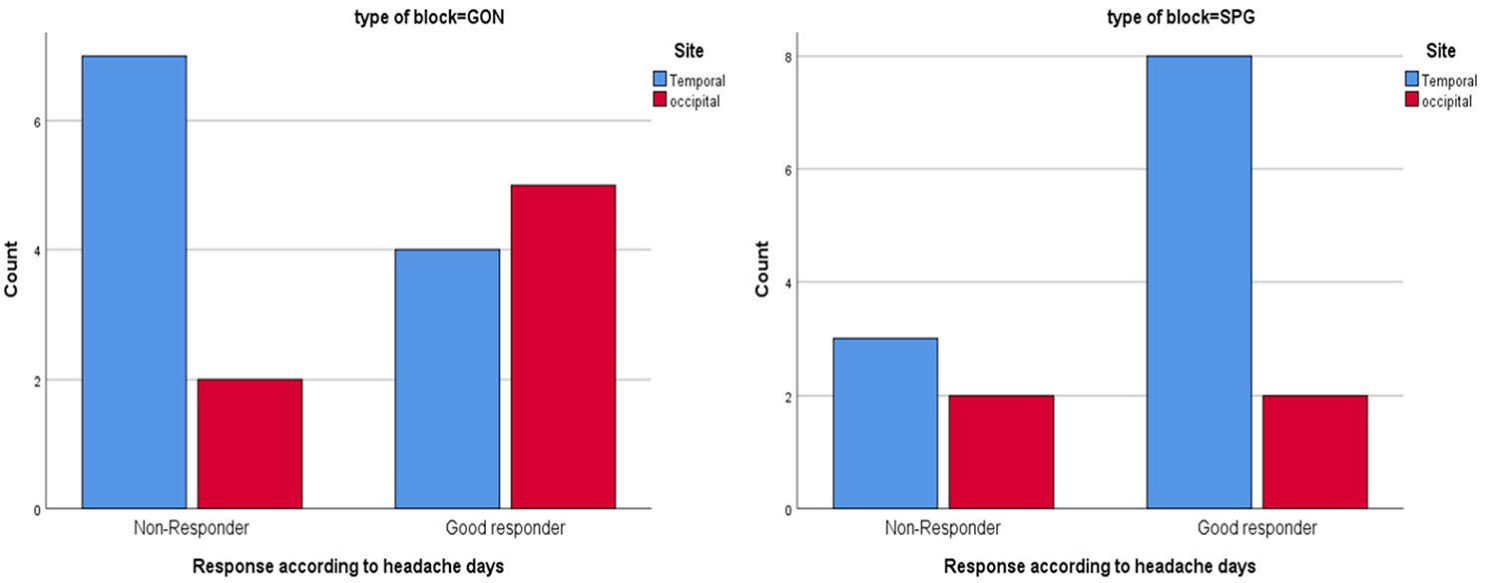
Responders rate among the two active groups in relation to site of pain. Difference in the response according to headache days in the GON group (left image) and the SPG group (right image) in relation to site of pain

The response to type of block was also compared according to the location of pain, and although that the number of good responders among those with temporal pain was higher among SPG group compared to GON group (72.7% vs. 26.4% respectively), and those with occipital pain showed better rate of response to GON compared to SPG (71.4% vs. 50% respectively), yet these differences were not statistically significant in either group (*p* = 0.56 and 0.335 respectively) (Table 4) (Fig. 6).

**Table 4:** Comparison of the response in relation to site of pain among the two groups

Type of block	Response		Tender points site		Fisher’s exact test
			Temporal	Occipital	p-value
GON	Response according to headache days	Non-Responder	7 (63.6%)	2 (28.6%)	0.335
		Good responder	4 (36.4%)	5 (71.4%)	
	Total		11 (100%)	7 (100%)	
SPG	Response according to headache days	Non-Responder	3 (27.3%)	2 (50%)	0.560
		Good responder	8 (72.7%)	2 (50%)	
	Total		11 (100%)	4 (100%)	

SPG: Sphenopalatine ganglion, GON: Greater Occipital nerve

### Safety

The side effects reported by 90% of all cases was local pain at the site of injection that lasted from 24 to 48 h. For GON group, patients suffered transient post-procedural dizziness lasting for few minutes due to positioning during the procedure. For the SPG group, there was local bleeding during the procedure in 80% of the cases, that was controlled by minimal compression for 2 min, and post-procedural pain reported by 40% of cases in the TMJ due to maintenance of mouth opening during injection. This was relieved by simple analgesics within 48 to 72 h. They also reported facial swelling at injection site (30% of cases) relieved by hot fomentations within next 24 h.

## Discussion

This study compared the efficacy and safety of ultrasound-guided SPG block and GON block in cases of chronic resistant migraine and showed that both types of nerve block were equally effective in improving the headache parameters (attack frequency, duration and intensity) and its impact on patients’ functionality and quality of life as assessed by HIT-6 and MIDAS scale. Despite that both types of block were equally effective, yet our results showed that patients with temporal pain or those suffering of autonomic manifestations showed better response to SPG block, while patients with occipital pain showed better response to GON block.

Ultrasound guided GON block at level of C2 was effective in improving all headache parameters in our study of chronic resistant patients. Abbas et al. similarly showed the efficacy of ultrasound guided GON block in a series of 40 chronic migraine patients on clinical parameters and CGRP levels [5].

Although distal GON block is more common, yet in our study we used the ultrasound-guided proximal block to ensure consistent delivery of the injectate directly to the greater occipital nerve (GON) in all patients, intending to minimize the impact of anatomical variations and reduce inter-operator differences, thereby enhancing the standardization and reproducibility of the technique throughout the study. Additionally, we hypothesized that targeting the GON at the proximal level, near its emergence from the C2 nerve root, might allow for a more effective blockade by achieving a broader spread of the injectate around the nerve trunk, potentially leading to better clinical outcomes compared to distal approaches. Notably, a recent study also demonstrated the feasibility, effectiveness and safety of the ultrasound-guided proximal GON block at the C2 level compared to distal block in migraine patients, further supporting the rationale for our chosen technique [21].

Also, another study conducted in Turkey in 2022 showed that proximal ultrasound guided GON block was as effective as distal block as a treatment modality for migraine patients, although they included episodic and chronic migraine patients and injected the recruited patients with bupivacaine bilaterally [6]. Furthermore, our study findings matched the study reported by *Viganò et al.* that assessed the effectiveness of GON block in chronic migraineurs on neurophysiological parameters by comparing the intensity dependant auditory evoked potentials slope (IDAP slope) pre and post treatment to healthy volunteers, as Auditory evoked potentials reflect central serotonin pathways, that was assumed to be disrupted in migraine patients leading to depressive symptoms. They showed a decrease in the steepness of the IDAP slope from baseline within 1 week of nerve block, reflecting elevation in serotonin firing, and a positive correlation to clinical improvement seen after 1 month of nerve block reflecting the role of nerve block in the modulating chronic migraine pathophysiology [22].

Our results are also consistent with a placebo-controlled trial that assessed distal GON block efficacy in chronic migraine patients and showed it to be effective compared to placebo. However, in our study we included resistant cases, and the patients were injected once during the study, compared to once/week for 4 successive weeks (4 sessions) in that study [23].

The current study also shows the effectiveness of ultrasound-guided SPG block through the infra-zygomatic approach, in improving all headache parameters with almost 50% reduction in number of headache days/month and reducing its functional impact significantly. This is similar to the results of a Turkish study in 2021 that assessed the effectiveness of SPG block for chronic resistant migraine patients, that showed significant reduction in the frequency, duration, and intensity of headache attacks over 8 weeks period, yet in our study the patients were injected once compared to 4 biweekly sessions through trans-nasal approach [8]. Moreover, the aforementioned study reported some side effects such as nasal bleeding, coughing, sneezing and discomfort during each session, with one case being excluded due to occurrence of vomiting during the procedure. The approach we used in the current study only caused local pain and swelling at site of injection that lasted around 24–48 h.

On the other hand, our study disagrees with another study by Cady et al. [24] that assessed the efficacy of SPG block through repeated trans-nasal approach sessions and showed no effect on either headache frequency, intensity or functional impact of migraine as assessed by HIT-6 scale compared to placebo. This contradictory result could be related to the approach of the procedure, as the ultrasound guided infra-zygomatic approach is a goal directed local injection, compared to the trans-nasal approach that depends on the diffusion of the drug from the applicator. Also, it could be related to the drugs being used in both studies, as in our study the block was achieved by lidocaine and steroids compared to bupivacaine only in that study.

Although the supra-zygomatic approach to SPG block may carry a lower risk of vascular adverse events, however, at our center, the infra-zygomatic approach is the standard technique routinely performed by our team, with extensive training and experience in its application. To further enhance safety, we performed the procedure under ultrasound guidance, which allowed for real-time visualization of surrounding vascular structures, and have not encountered any serious adverse events related to this approach in our clinical practice.

Studies that assessed the efficacy of GON block compared to placebo showed better response to the nerve block as showed in a narrative review about GON block in migraine prophylaxis that concluded that GON block is effective as preventive treatment for chronic migraine [25] and since our results showed a lack of superiority of GON over SPG block, therefore ultrasound guided SPG block may be an alternative in certain cases.

One study previously compared the efficacy of GON block to SPG block in chronic migraine [26]. They used the trans-nasal approach over 4 weekly sessions and similarly showed no difference between groups despite both blocks having a good response rate in reducing clinical parameters.

Also, another study that compared the efficacy of GON block and SPG block in episodic migraine [27], were patients also received the block weekly over 4 weeks then once/month for 2 months and showed better response to GON block in the 3rd month. This could be attributed to using the trans-nasal approach in the SPG block.

Moreover, our study was concerned with resistant cases, being a challenge in medical practice, aiming to eventually provide a feasible cost-effective alternative or adjunct to other more expensive approved lines of treatment as anti-CGRP and botulinum toxin A.

The trend for higher responder rate towards the SPG in cases with autonomic manifestations, could be explained by the SPG being part of the parasympathetic outflow responsible for the autonomic symptoms during the migraine attack, as evidenced by the effectiveness of SPG block in cases of trigeminal autonomic cephalalgia (TACs)/cluster headache [28, 29].

Our study is limited by the small number of patients. A larger sample may allow for identifying further subgroups of patients who could benefit from each technique. We have taken steps to ensure blinding of the patient to the treatment modality yet blinding of the physician was not feasible.

## Conclusion

Both GON block and SPG block were effective in improving patients with chronic resistant migraine, with significant and clinically-meaningful reduction in headache frequency, intensity and in reducing functional impairment. There were no significant differences in efficacy between the two techniques and both were reasonably safe. Some patients may benefit more from SPG block, particularly those with cranial autonomic symptoms and with temporal location of pain.

## Data Availability

No datasets were generated or analysed during the current study.

## Abbreviations

CM: Chronic Migraine
GON: Greater Occipital Nerve
SPG: Sphenopalatine Ganglion
NRS: Numerical Rating Scale
HIT-6: Headache Impact test-6
MIDAS: Migraine Disability Assessment Scale
TMJ: Temporomandibular Joint
TACs: Trigeminal Autonomic Cephalalgias
IDAP: Intensity dependant auditory evoked potentials

## References

[1] Soto E, Bobr V, Bax JA (2012) Interventional techniques for headaches. Tech Reg Anesth Pain Manag 16:30–40. 10.1053/j.trap.2012.11.005

[2] Sacco S, Braschinsky M, Ducros A, Lampl C, Little P, van den Brink AM, Pozo-Rosich P, Reuter U, de la Torre ER, Sanchez, Del Rio M, Sinclair AJ, Katsarava Z, Martelletti P (2020) European headache federation consensus on the definition of resistant and refractory migraine. J Headache Pain 21:76. 10.1186/s10194-020-01130-5 10.1186/s10194-020-01130-5 PMC7296705 32546227

[3] Ashkenazi A, Blumenfeld A, Napchan U, Narouze S, Grosberg B, Nett R, DePalma T, Rosenthal B, Tepper S, Lipton RB (2010) Peripheral nerve blocks and trigger point injections in headache management - A systematic review and suggestions for future research. Headache 50:943–952 20487039 10.1111/j.1526-4610.2010.01675.x

[4] Zhang H, Yang X, Lin Y, Chen L, Ye H (2018) The efficacy of greater occipital nerve block for the treatment of migraine: A systematic review and meta-analysis. Clin Neurol Neurosurg 165:129–133 29421172 10.1016/j.clineuro.2017.12.026

[5] Abbas A, Moustafa R, Shalash A, Haroun M, Amin R, Borham S, Elsadek A, Helmy S (2022) Serum CGRP changes following Ultrasound-Guided bilateral Greater-Occipital-Nerve block. Neurol Int 14:199–206. 10.3390/neurolint14010016 35225886 10.3390/neurolint14010016 PMC8883968

[6] Karaoğlan M, İnan LE (2022) A comparison of the clinical efficacy of GON block at the C2 level and GON block at the classical distal occipital level in the treatment of migraine. Clin Neurol Neurosurg 215. 10.1016/j.clineuro.2022.107190 10.1016/j.clineuro.2022.107190 35286995

[7] Boezaart A, Smith C, Reyneke J (2018) Pterygopalatine ganglion block: for effective treatment of migraine, cluster headache, postdural puncture headache and postoperative pain, 1st edn. Amazon digital Services LLC

[8] Tepe N, Tertemiz OF (2021) The effectiveness of Sphenopalatine ganglion Blockade in chronic migraine resistant to medical treatment. Neurol Asia 26:737–741. 10.54029/2021JXN

[9] Mehta D, Leary MC, Yacoub HA, El-Hunjul M, Kincaid H, Koss V, Wachter K, Malizia D, Glassman B, Castaldo JE (2019) The effect of regional anesthetic Sphenopalatine ganglion block on Self-Reported pain in patients with status migrainosus. Headache 59:69–76. 10.1111/head.13390 30043973 10.1111/head.13390

[10] Tolba R, Weiss AL, Denis DJ (2019) Sphenopalatine ganglion block and radiofrequency ablation: technical notes and efficacy. Ochsner J 19:32–37 30983899 10.31486/toj.18.0163 PMC6447206

[11] Tepper SJ, Stillman MJ (2013) Cluster headache: potential options for medically refractory patients (when all else fails). Headache 53:1183–1190 23808603 10.1111/head.12148

[12] Anugerah A, Nguyen K, Nader A (2020) Technical considerations for approaches to the ultrasound-guided maxillary nerve block via the pterygopalatine fossa: a literature review. Regional Anesthesia & Pain Medicine 45:301. 10.1136/rapm-2019-100569 10.1136/rapm-2019-100569 31924742

[13] Jensen MP, McFarland CA (1993) Increasing the reliability and validity of pain intensity measurement in chronic pain patients. Pain 55 10.1016/0304-3959(93)90148-I 8309709

[14] Ailani J, Andrews JS, Rettiganti M, Nicholson RA (2020) Impact of galcanezumab on total pain burden: findings from phase 3 randomized, double-blind, placebo-controlled studies in patients with episodic or chronic migraine (EVOLVE-1, EVOLVE-2, and REGAIN trials). J Headache Pain 21. 10.1186/s10194-020-01190-7 10.1186/s10194-020-01190-7 PMC7568830 33069214

[15] Kosinski M, Bayliss MS, Bjorner JB, Ware JE, Garber WH, Batenhorst A, Cady R, Dahlöf CGH, Dowson A, Tepper S (2003) A six-item short-form survey for measuring headache impact: the HIT-6™. Qual Life Res 12:963–974. 10.1023/A:1026119331193 14651415 10.1023/a:1026119331193

[16] Stewart WF, Lipton RB, Kolodner KB, Sawyer J, Lee C, Liberman JN (2000) Validity of the migraine disability assessment (MIDAS) score in comparison to a diary-based measure in a population sample of migraine sufferers. Pain 88 10.1016/S0304-3959(00)00305-5 11098098

[17] Mourad D, Hajj A, Hallit S, Ghossoub M, Khabbaz L (2019) Validation of the Arabic version of the migraine disability assessment scale among Lebanese patients with migraine. J Oral Facial Pain Headache 33:47–53. 10.11607/ofph.2102 30153314 10.11607/ofph.2102

[18] Togha M, Jafari E, Moosavian A, Farbod A, Ariyanfar S, Farham F (2021) Cranial autonomic symptoms in episodic and chronic migraine: a cross sectional study in Iran. BMC Neurol 21. 10.1186/s12883-021-02513-0 10.1186/s12883-021-02513-0 PMC8686360 34930166

[19] Andreou AP, Edvinsson L (2019) Mechanisms of migraine as a chronic evolutive condition. J Headache Pain 20. 10.1186/s10194-019-1066-0 10.1186/s10194-019-1066-0 PMC6929435 31870279

[20] Arab A, Khoshbin M, Karimi E, Saberian G, Saadatnia M, Khorvash F Effects of greater occipital nerve block with local anesthetic and triamcinolone for treatment of medication overuse headache: an open-label, parallel, randomized, controlled clinical trial. 10.1007/s10072-021-05295-y/Published 10.1007/s10072-021-05295-y 33945036

[21] Gürsoy G, Tuna HA (2024) Comparison of two methods of greater occipital nerve block in patients with chronic migraine: ultrasound-guided and landmark-based techniques. BMC Neurol 24. 10.1186/s12883-024-03816-8 10.1186/s12883-024-03816-8 PMC11373286 39232647

[22] Viganò A, Torrieri MC, Toscano M, Puledda F, Petolicchio B, Sasso D’Elia T, Verzina A, Ruggiero S, Altieri M, Vicenzini E, Schoenen J, Di Piero V (2018) Neurophysiological correlates of clinical improvement after greater occipital nerve (GON) block in chronic migraine: relevance for chronic migraine pathophysiology. J Headache Pain 19. 10.1186/s10194-018-0901-z 10.1186/s10194-018-0901-z PMC6102162 30128946

[23] Gul HL, Ozon AO, Karadas O, Koc G, Inan LE (2017) The efficacy of greater occipital nerve Blockade in chronic migraine: A placebo-controlled study. Acta Neurol Scand 136:138–144. 10.1111/ane.12716 27910088 10.1111/ane.12716

[24] Cady RK, Saper J, Dexter K, Cady RJ, Manley HR (2015) Long-term efficacy of a double-blind, placebo-controlled, randomized study for repetitive Sphenopalatine Blockade with bupivacaine vs saline with the Tx360^®^ device for treatment of chronic migraine. Headache 55:529–542. 10.1111/head.12546 25828648 10.1111/head.12546 PMC6681144

[25] Inan LE, Inan N, Unal-Artık HA, Atac C, Babaoglu G (2019) Greater occipital nerve block in migraine prophylaxis: narrative review. Cephalalgia 39:908–920 30612462 10.1177/0333102418821669

[26] Balta S, Uca AU, Odabas FO, Demir A (2023) Comparison of outcomes of transnasal Sphenopalatine ganglion and ultrasound-guided proximal greater occipital nerve Blockades in chronic migraine. Neurol Asia 28:337–347. 10.54029/2023dpf

[27] Unal HA, Basarı A, Celiker OS, Cakar Turhan KS, Asik I, Ozgencil GE (2024) Comparison of greater occipital nerve Blockade and Sphenopalatine ganglion Blockade in patients with episodic migraine. J Clin Med 13. 10.3390/jcm13113027 10.3390/jcm13113027 PMC11173077 38892738

[28] Costa A, Pucci E, Antonaci F, Sances G, Granella F, Broich G, Nappi G (2000) The effect of intranasal cocaine and Lidocaine on Nitroglycerin-Induced attacks in cluster headache. Cephalalgia 20:85–91. 10.1046/j.1468-2982.2000.00026.x 10961763 10.1046/j.1468-2982.2000.00026.x

[29] Narouze S, Kapural L, Casanova J, Mekhail N (2009) Sphenopalatine ganglion radiofrequency ablation for the management of chronic cluster headache. Headache: J Head Face Pain 49:571–577. 10.1111/j.1526-4610.2008.01226.x 10.1111/j.1526-4610.2008.01226.x 18783451

